# In this month’s Bulletin

**DOI:** 10.2471/BLT.26.000526

**Published:** 2026-05-01

In the editorial section, Ritu Sadana et al. (286) call for papers for a special theme issue on a life course approach to health and well-being. Lorenzo Moja et al. (287) encourage national governments to increase alignment with WHO’s *Essential medicines list* to mark a 50-year anniversary of this guidance.

## Egypt

### Recommendations tailored for context

Mehrnaz Kheirandish et al. (350–356) describe the development of a national guideline adaptation programme.

## India

### Hepatitis A virus endemicity 

Avinash R Deoshatwar et al. (290–300) estimate seroprevalence in eight states. 

## the Gambia

### Antenatal testing for hepatitis B prophylaxis eligibility

Alassane Ndiaye et al. (301–314) compare carbon emissions generated by three diagnostic methods.

## Global 

### The compilation of a global traditional medicine library

Carmen VM Abdala et al. (328–341) map online sources of knowledge on traditional, complementary and integrative medicine.

### Negative externalities of health research

Gabrielle Samuel and Federica Lucivero (342–349) examine ways of measuring environmental harms.

### Health-related sustainable development goal indicators 

Keyrellous Adib et al. (315–327) evaluate the extent of missing data.

### Research on health and climate change

Soumyadeep Bhaumik (357–359) discusses just approaches to priority-setting.

### Mental health of parents 

Indrani Paul et al. (360–362) consider the need for support during early childhood.

### A global focus on pre-eclampsia 

Jeremy Farrar et al. (363–365) present a shared commitment to prevention, detection and treatment.

### WHO recommendations for pregnancy and postpartum care 

Andrea Solnes Miltenburg et al. (366–368) note a need to cover hypertensive disorders of pregnancy. 

